# Shoulder Impingement, An Uncommon Complication of Distal Clavicle Fracture Treated Arthroscopically: A Case Report

**DOI:** 10.5704/MOJ.1311.002

**Published:** 2013-11

**Authors:** CS Wang, KC Bea, O Zulkiflee

**Affiliations:** Department of Orthopaedics, Penang General Hospical , Georgetown , Malaysia; Department of Orthopaedics, Penang General Hospical , Georgetown , Malaysia; Department of Orthopaedics, Penang General Hospical , Georgetown , Malaysia

## Abstract

Clavicle fracture is commonly treated conservatively. However uncommon complication can arise causing impingement. We report a patient who sustained distal clavicle fracture and was treated conservatively. However he developed persistent shoulder pain that affected his daily life. Shoulder impingement was diagnosed and arthroscopic subacromioclavicular decompression was done. Following early physiotherapy the early recovery was good with full range of motion of the shoulder.

**Key Words:**

Clavicle fracture, shoulder impingement, arthroscopic subacromioclavicular decompression

## Introduction

Clavicle is one of the most frequently fractured bones in motor vehicle accidents. In young individuals clavicle fracture contributes to approximately 2.6% of all fractures^2^. The significant associated injuries must be ruled out like chest and head trauma. The nonoperative treatment is simple, by applying a standard arm sling as studies have shown that other options like body jacket, cast, brace or figure-of-eight bandage are not superior^2^. The undisplaced fractures of diaphysis or the lateral end of clavicle has a high union rate and the functional outcomes are good when treated conservatively^4^,^4^. Most adult clavicle fractures heal with minimal persistent symptoms or none at all. The potential problems include non-union and malunion of the fractured clavicle, and rarely shoulder impingement^2^. These patients experience symptoms when they abduct the affected shoulder or elevate the arm to the overhead position. We report our experience in treating - arthroscopically a patient with shoulder impingement after conservative management of a distal clavicle fracture.

## Case Report

A 36 years old gentleman who was involved in a motor vehicle accident was seen at a hospital. He was diagnosed with a closed fracture of the right clavicle and given an arm sling before an appointment to the Orthopaedic clinic. At review at the Orthopaedic clinic three weeks after the trauma, he was started on physiotherapy and discharged from further followup. A year post trauma, he was referred by the out patient department to our center for further management in view of persistent shoulder pain and reduced range of movement despite the fracture having healed, and inability to resume his job.

**Fig. 1:**
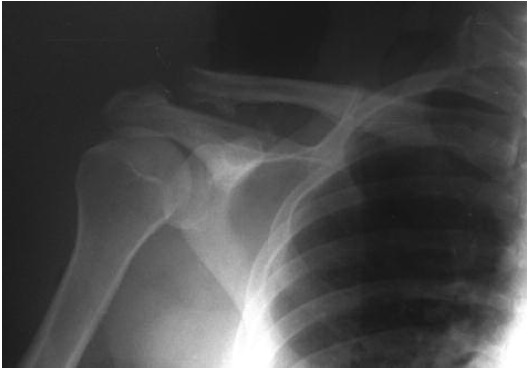
: Radiograph showing bony spur followingdistal clavicle fracture.

On examination Hawkins test was positive. There was no shoulder deformity or point tenderness noted. Active range of motion of the right shoulder was reduced due to pain, with active flexion limited to 90 degrees and abduction to 70 degrees. The passive range of movement was full. The right shoulder radiograph revealed a beak-shaped osteophyte at the fracture site of an Allman type II united clavicle fracture. (Figure 1)

The diagnosis of subacromioclavicular joint bony impingement secondary to fracture bony callus spur was established. He underwent arthroscopic subacromioclavicular decompression. Intraoperatively we observed that the supraspinatous was intact. The subacromioclavicular osteophyte was observed (Figure 3a) and resected so that the shoulder was free from impingement in all ranges of motion (Figure 3b).

**Fig. 2:**
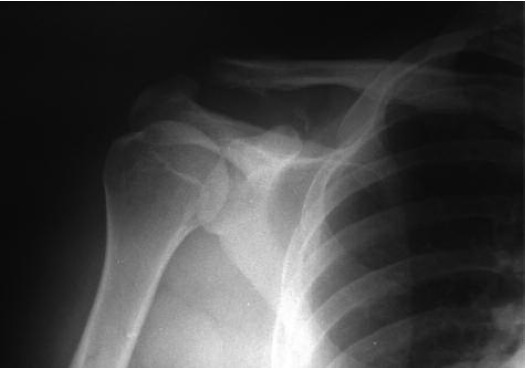
: Radiograph taken after arthroscopic subacromioclavicular decompression, bony spur resected.

Early rehabilitation was started on the next day of surgery with physiotherapy and pain relief medications. The postoperative x-ray showed spacious subacromioclavicular space (Figure 2). He was discharged well from hospital a day later.

Subsequently he was followed up at the Orthopaedic clinic at two weeks duration for the sutures to be removed. During the visit his preoperative pain was relieved and he regained full range of motion in the affected shoulder: the active flexion and abduction improved to 180 degrees (Figure 4a, 4b). Outpatient physiotherapy was continued and he was followed up for a year without signs of recurrence.

## Discussion

Distal clavicle fracture is commonly treated non-operatively, where the uncommon delayed complication of impingement syndrome is often not recognised. The symptomatic cases with non-union or malunion have range from three to five percents in a number of reports.

However data is lacking on the incidence of shoulder impingement following distal clavicle fracture1. Kuriyama and Inoue reported a similar case treated with arthroscopic decompression.

This rare complication following conservative management of distal end clavicle fracture is infrequent^1^ and easily missed especially by doctors in busy follow-up clinics where these patients are usually discharged prematurely post injury. There is also no established guideline for the duration of follow up for patients with clavicle fractures. The diagnosis in this case had been delayed, as the patient was managed with continuation of analgesics and physiotherapy when he complained of persistent pain. It was a disabling problem for the patient and he could not resume his job.

Careful examination with a plain shoulder radiograph, preoperative planning and counselling, precise removal of the impinging subacromioclavicular osteophyte and meticulous observation of postoperative physiotherapy of the shoulder yield successful results. Standard portals arthroscopic subacromioclavicular decompression in our patient produced a favourable result mainly with dramatic increase in the active range of motion and significant relief of preoperative pain with improvement in shoulder function.

Good or excellent results (74-93%) have been reported in arthroscopic subacromial decompression for shoulder impingements^5^. In young and active patients with subacromial impingement syndrome the arthroscopic method is the gold standard^5^. However the diagnosis of underlying cause of shoulder impingement must be clearly established.

**Fig. 3a:**
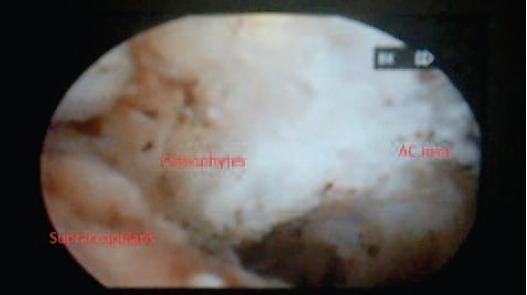
: Arthroscopic view showing the bony spur at subacromioclavicular space.

**Fig. 3b:**
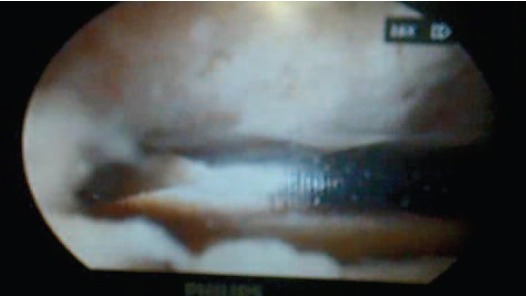
: Arthroscopic subacromioclavicular decompression procedure showing resection of bony spur.

**Fig. 4a:**
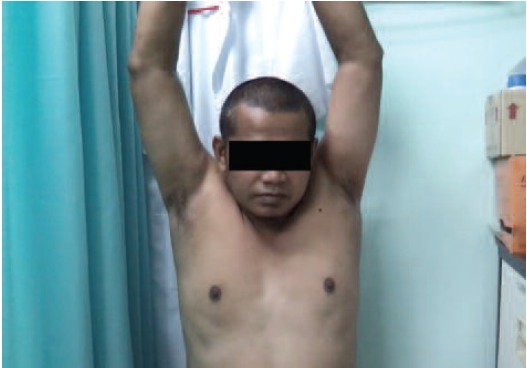
: Full abduction of the right shoulder on follow up at the clinic.

**Fig. 4b:**
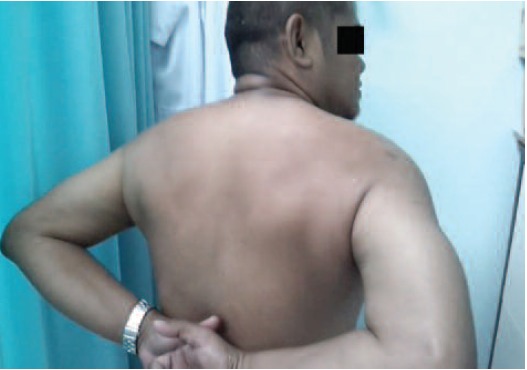
: Good range of motion of the right shoulder with good functional outcome.

## Conclusion

Shoulder impingement is an uncommon complication of distal end clavicle fracture. Careful clinical assessment is required to establish the diagnosis to prevent delayed treatment. Arthroscopic decompression is a recommended method of treatment and shows good early clinical outcome.
